# The Impact of ABO Blood Grouping on COVID-19 Vulnerability and Seriousness: A Retrospective Cross-Sectional Controlled Study among the Arab Community

**DOI:** 10.3390/ijerph18010276

**Published:** 2021-01-01

**Authors:** Nagla A. El-Shitany, Manal El-Hamamsy, Ahlam A. Alahmadi, Basma G. Eid, Thikryat Neamatallah, Haifa S. Almukadi, Rana A. Arab, Khadija A. Faddladdeen, Khayria A. Al-Sulami, Safia M. Bahshwan, Soad S. Ali, Steve Harakeh, Shaimaa M. Badr-Eldin

**Affiliations:** 1 Department of Pharmacology and Toxicology, Faculty of Pharmacy, King Abdulaziz University, Jeddah 21589, Saudi Arabia; beid@kau.edu.sa (B.G.E.); Taneamatallah@kau.edu.sa (T.N.); hsalmukadi@kau.edu.sa (H.S.A.); 2 Department of Pharmacology and Toxicology, Faculty of Pharmacy, Tanta University, Tanta 31527, Egypt; 3 Department of Pharmacy Practice, Faculty of Pharmacy, King Abdulaziz University, Jeddah 21589, Saudi Arabia; melhamamsy@kau.edu.sa; 4 Department of Clinical Pharmacy, Faculty of Pharmacy, Ain Shams University, Cairo 11566, Egypt; 5 Department of Biological Sciences, Faculty of Science, King Abdulaziz University, Jeddah 21589, Saudi Arabia; aahmadi1000@hotmail.com (A.A.A.); kfaddladdeen@kau.edu.sa (K.A.F.); 6 Medicine Program, Ibn Sina National Faculty for Medical Studies, Jeddah 22421, Saudi Arabia; charmingpearl1996@hotmail.com; 7 Department of Biology, Faculty of Science and Arts in Al-Makhwah, Al-Baha University, Al-Baha 65511, Saudi Arabia; Alsulamikhayria@gmail.com; 8 Department of Biology, Faculty of Science, Al-Baha University, Al-Baha 65511, Saudi Arabia; Safia_bahshwan@yahoo.com; 9 Department of Histology, Faculty of Medicine, Assiut University, Assiut 71515, Egypt; soadshaker@gmail.com; 10 Special Infectious Agents Unit, King Fahd Medical Research Center, Yousef Abdullatif Jameel Chair of Prophetic Medicine Application, Faculty of Medicine, King Abdulaziz University, Jeddah 21589, Saudi Arabia; sharakeh@gmail.com; 11 Department of Pharmaceutics and Industrial Pharmacy, Faculty of Pharmacy, King Abdulaziz University, Jeddah 21589, Saudi Arabia; Sbadr5@hotmail.com; 12 Department of Pharmaceutics and Industrial Pharmacy, Faculty of Pharmacy, Cairo University, Cairo 11562, Egypt

**Keywords:** COVID-19, blood group, artificial respiration, oxygen saturation, myalgia

## Abstract

*Background and Objectives:* Studies have noted that some ABO blood types are more susceptible to COVID-19 virus infection. This study aimed to further confirm the relationship between different blood groups on the vulnerability, symptoms, cure period, and severity among COVID-19 recovered patients. *Subjects and Methods:* This cross-sectional study approached the participants from the Arab community via social media (mainly Facebook and WhatsApp). The data were collected through two Google Form questionnaires, one for COVID-19 recovered patients (COVID-19 group, *n* = 726), and the other for the healthy people (Control group, *n* = 707). *Results:* The subjects with blood group O were the least likely to be infected with the COVID-19 virus, while those with blood group A were not likely to be the most susceptible. There were significant differences among different ABO blood groups regarding the distribution of oxygen saturation percentage, myalgia, and recovery time after COVID-19 infection (*p* < 0.01, 0.01, and 0.05, respectively). The blood group A showed the highest percentage of patients who experienced an oxygen saturation range of 90–100%, whereas the blood group O showed the highest percentage of patients who experienced an oxygen saturation range of 70–80%. The blood group A showed the lowest percentage of patients who required artificial respiration, whereas the blood group O showed the highest percentage of patients who required artificial respiration. The blood group B showed the lowest percentage of patients who experienced myalgia and exhibited the lowest percentage of patients who needed 3 weeks or more to recover. *Conclusion:* The people of blood group O may be the least likely to be infected with COVID-19, however, they may be the more in need of treatment in hospital and artificial respiration compared to the other blood groups.

## 1. Introduction

The novel coronavirus disease (COVID-19) is caused by enveloped RNA viruses (SARS-CoV-2). SARS-CoV-2 viruses (order Nidovirales, family Coronaviridae, and subfamily Orthocoronavirinae) are mostly spherical, given electron microscopic morphology, although some are polygonal. The virus is approximately 125 nm in diameter and is enveloped with spikes (glycoproteins) that are nearly 9 to 12 nm, creating the virus′s coronal form. The virus morphology is compatible with others in the coronavirus family, particularly SARS-CoV and MERS-CoV [1,2]. The target cell receptor is the key to determining how the virus reaches the cell and which tissues are targeted. The spike glycoproteins engage the viral envelope with the targeted cell cytomembrane. Current research studies showed that angiotensin-converting enzyme 2 (ACE2) is expected to be the COVID-19 cell receptor [1].

The COVID-19 virus causes a severe respiratory infection associated with a considerable mortality rate. This rapidly spreading viral infection was proclaimed a pandemic by the World Health Organization (WHO) in March 2020 [3]. The wide spread of COVID-19 might be explained by its human-to-human transmission not only from symptomatic patients but also from asymptomatic and pre-symptomatic infected persons [4].

The major clinical signs of COVID-19 in humans include fever, cough, fatigue, anorexia, myalgia, and diarrhea. The disease usually progresses from mild to severe illness in approximately one week following the start of the symptoms. Dyspnea is the most prevalent symptom of severe disease and is often associated with hypoxemia [5]. Most patients suffering from aggravated COVID-19 exhibit lymphopenia, and some of them develop central and peripheral nervous system disorders. Severe COVID-19 infection may also result in acute cardiac, renal, and hepatic damage. Rhabdomyolysis, coagulopathy, and shock were also reported in severe cases [6]. COVID-19 subjects who were admitted to the intensive care unit (ICU) and had a need for mechanical ventilation or a fraction of inspired oxygen (Fi O2) of 60% were recognized as critically ill patients [7].

Risk factors for death occurrence and susceptibility of patients infected with the SARS-CoV-2 virus included age, gender, obesity, and other chronic diseases. The diagnosis of COVID-19 can be performed based on the viral RNA detection in respiratory secretions along with clinical background. The detection of COVID-19 virus can be affirmed by a positive reverse transcriptase polymerase-chain-reaction (PCR) test on nasal or pharyngeal swabs from suspected individuals. Besides, bilateral consolidations and/or ground-glass opacities shown in chest radiographs are common in COVID-19-infected patients. Thus, radiographs could also be considered as a valuable indicator for COVID-19 infection [8].

Individuals with particular ABO blood groups are more prone to various types of infections [9]. For example, blood types A and AB predispose subjects to aggravated malaria, while type O causes resistance to several protozoal infections. Furthermore, this blood grouping system exhibit direct or indirect association with some cardiovascular conditions [10]. Recently, Groot et al. reported that people with A, B, and AB blood types are more susceptible to thrombosis and myocardial infarction, while those bearing the O blood group are more prone to hypertension [11]. In addition, individuals having the A antigen are also predisposed to a higher risk of metabolic disorders, such as hyperlipidemia and diabetes mellitus [12].

After the outbreak of the COVID-19 infection, the likelihood of association between ABO blood groups and the susceptibility to COVID-19 exposure has been reported in patients from three hospitals in Wuhan, Shenzhen, and China. The study results showed that individuals with blood group A had a markedly greater risk of COVID-19 exposure, whereas those with blood group O had a significantly reduced risk of COVID-19 infection. However, the researchers recommended more studies to confirm the association between the ABO blood grouping and COVID-19 viral infection [13]. In a meta-analysis of two different case-control cohorts, type A blood was reported to confer a greater risk of aggravated COVID-19, while type O blood may offer protection against COVID-19 infection [14].

In the face of this challenging pandemic, the current retrospective cross-sectional study aimed at investigating the influence of ABO blood grouping type on the vulnerability, symptoms, cure period, and severity among COVID-19 recovered patients compared to a control group.

## 2. Subjects and Method

### 2.1. Study Protocol

This was a retrospective cross-sectional study conducted on participants from Saudi Arabia and Egypt. The study was carried out over two weeks, from the 17th until the 31st of August 2020. The study protocol was approved by the Unit of Biomedical Ethics Research Committee, Faculty of Medicine, King Abdulaziz University, Saudi Arabia (Reference No. 65920). Two Google Form questionnaires were designed and written in the Arabic language, one for the COVID-19-recovered patients (COVID-19 group), and the other one for the healthy people (Control group). The questionnaires were distributed via social media (mainly Facebook and WhatsApp). Communication between the researchers and the participants was conducted by electronic mail when needed. The COVID-19 questionnaire comprised two categories of questions. The first category covers background information of the participant, such as age group, gender, nationality, blood group, and Rhesus factor (Rh). The second category of questions inquired about potential exposure of the participant to COVID-19 and focused on the symptoms that appeared and their duration, such as fever degree, oxygen saturation, medications taken, and whether the participant was treated at home or admitted to the hospital. The control questionnaire included questions similar to that of the first category of questions in the COVID-19 questionnaire, but not the second. All participants were allowed to terminate the survey at any time. All measures were taken to keep the confidentiality of the data. The survey was outlined following the pertinent guidelines and regulations of the National Committee of Bioethics, Saudi Arabia.

### 2.2. Inclusion Criteria

Inclusion criteria for the COVID-19 group included the following: previous infection with the virus, complete recovery, and knowledge of blood group type. The inclusion criteria for the control group included the following: no previous infection with the COVID-19 virus and knowledge of blood group type.

### 2.3. Exclusion Criteria

Questionnaires that included a negative diagnostic smear for COVID-19 (negative PCR test) without any other approved diagnostic method such as complete blood picture, serum ferritin, diagnostic chest X-ray, or even the absence of more than two distinct COVID-19 symptoms were excluded.

### 2.4. Sample Size

**Figure 1:**
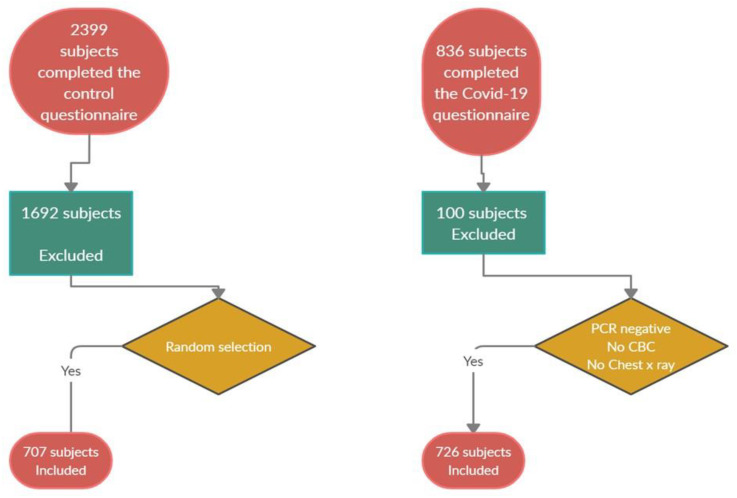
Flowchart for the Covid-19 and control questionnaires.

The minimum sample size for conducting this survey was 664 with a 1% margin of error and a 99% confidence level. In this study, 836 subjects completed the COVID-19 questionnaire. The number of questionnaires that met the inclusion and exclusion criteria reached 726. Furthermore, 2399 subjects completed the questionnaire for the control group; 707 of them were randomly selected for comparing results (Figure 1).

#### Study Variables

Background information, including gender, age, and nationality, in addition to ABO blood group and Rh factor, were investigated as independent variables. Exposure to COVID-19, symptoms, fever degree, oxygen saturation, and medications were considered responses.

### 2.5. Statistical Analysis

Descriptive statistics were applied for the background information. The responses were presented as counts and/or percentages. A chi-squared test was used for statistical analysis. The comparison between groups was done by calculating the standardized residuals online (http://vassarstats.net/newcs.html) and transforming the z-score to Bonferroni *p*-value online (https://www.calculator.net/z-score-calculator.html). The data were statistically analyzed using Prism^®^ (version 8.4.0, GraphPad Software Inc., La Jolla, CA, USA). The significant difference was considered at *p* ≤ 0.05.

## 3. Results

### 3.1. Demographic Characteristics and Blood Grouping of the Study Population

The control questionnaire was filled out by 707 subjects who were not previously infected with the COVID-19 virus. The control group subjects included 699 Egyptians, 2 Saudis, and 6 of other nationalities. Moreover, the COVID-19 questionnaire was filled out by another 726 subjects who had recovered from COVID-19. Those subjects, representing the COVID-19 group, included 700 Egyptians, 20 Saudis, and 6 of other nationalities. Out of the control and COVID-19 groups, the number of females was 590 (83.5%) and 616 (84.8%), respectively, while the number of males was 117 (16.5%) and 110 (15.2%), respectively (Table 1).

The study population fell into four different age groups, as shown in Table 1. Pearson chi-square test revealed a significant difference between the distribution of subjects in the diverse age groups in both the control and the COVID-19 groups (*p* < 0.001). According to the standardized residuals calculation for the age groups over 20 years, a nearly similar number of subjects was found in each class, with the majority lying in the 20–40 years group (565, or 79.9%, in the control group and 596, or 82.1%, in COVID-19 group). On the other hand, the number of subjects in the COVID-19 group below 20 years (9, 1.3%) was significantly lower compared to the control group (38, 5.4%) (Standardized residuals of 3.08 and 3.04, respectively; Bonferroni corrected *p*-value < 0.01).

COVID-19 viral infection was diagnosed through a positive PCR swab in 278 (38.3%) subjects. Simultaneously, the smear appeared negative with the existence of the characteristic symptoms, chest X-ray, neutropenia, and elevated serum ferritin in 157 subjects (21.6%). The remaining 291 (40.1%) subjects were not swabbed and were diagnosed with biochemical and clinical symptoms (Table 1).

### 3.2. Association between COVID-19 Virus Infection and the ABO Blood Group

The ABO blood group distribution in the study population was compiled in Table 1. In the control group, the percentage of distribution of subjects were 36%, 29%, 23%, and 12% for blood groups O, A, B, and AB, respectively. On the other hand, in the COVID-19 group, an ABO allocation of 28%, 35%, 25%, and 12% was presented for blood groups O, A, B, and AB, respectively. Pearson chi-squared test analysis revealed a significant difference among the distribution of ABO blood groups in both the control group and the COVID-19 group (*p* < 0.01). The percentage of the blood group O in the COVID-19 group was significantly lower than that in the control group (204, or 28%, versus 253, 36%) (Standardized residuals of 1.81 and 1.83, respectively, and Bonferroni corrected *p*-value < 0.05). On the other hand, the percentage of the blood group A in the COVID-19 group was insignificantly higher compared to the control group (258, or 35%, versus 207, 29%) (Standardized residuals of 1.46 and 1.48, respectively, and Bonferroni corrected *p*-value = 0.072 and 0.069, respectively). This study showed that the subjects with blood group type O were the least susceptible to COVID-19, while those subjects with blood group type A are most at risk.

The Rh distribution in the study population is shown in Table 1. In the control group, a percentage distribution of 16% and 84% is presented for Rh negative and Rh positive, respectively, while in the COVID-19 group, an Rh allocation of 18.6% and 81.4% for Rh negative and Rh positive is respectively presented. Pearson chi-squared test analysis revealed a non-significant difference between both Rh types in both the control group and the COVID-19 group (*p* = 0.1913).

### 3.3. Distribution of the Common Symptoms Recorded among the COVID-19 Group

**Figure 2:**
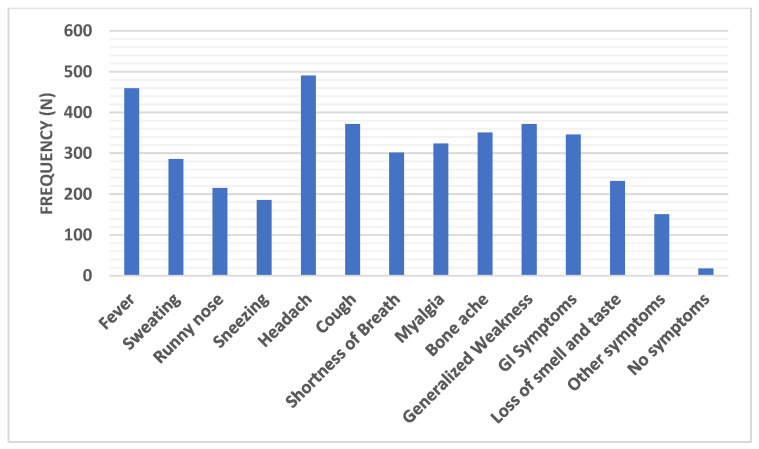
Distribution of the common symptoms among COVID-19 group. Data were presented as frequency (number).

The most common symptoms experienced by those with COVID-19 were headache in 490 subjects (67.5%), fever in 459 (63.2%), cough in 372 (51.2%), generalized weakness in 372 (51.2%), bone ache in 351 (48.3%), gastrointestinal discomfort (GI) in 346 (47.7%), myalgia in 324 (44.6%), and shortness of breath in 302 (41.6%). Less common symptoms included sweating in 286 (39.4%), loss of smell and taste in 232 (32%), a runny nose in 215 (29.6%), and sneezing in 186 (25.6%). There were 151 (20.8%) subjects who reported other symptoms (not mentioned) and 18 (2.5%) subjects reported no symptoms at all (Figure 2).

### 3.4. Distribution of Recovery Periods Recorded among the COVID-19 Group

**Figure 3:**
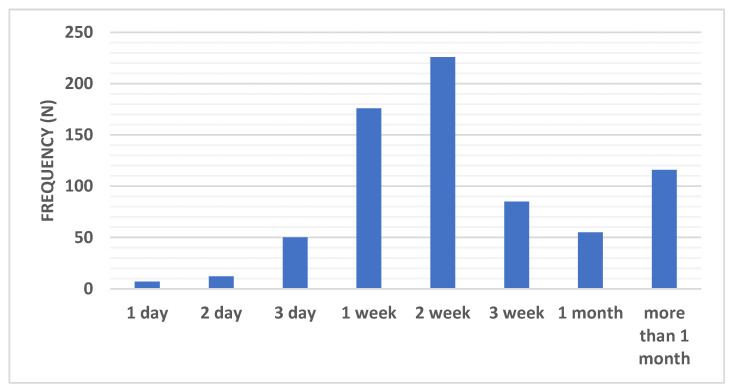
Distribution of recovery periods recorded among the COVID-19 group. Data were presented as frequency (number).

The results showed that most COVID-19 patients needed one to two weeks to recover from their symptoms (24.2% and 31.1%, respectively), while 16% of patients required more than a month to recover. Some patients recovered after 3 weeks (11.7%) or one month (7.5%). Small percentages of patients required a day (1%), two (1.6%), or three days (6.8%) to recover (Figure 3).

### 3.5. Distribution of Different Treatment Regimens among the COVID-19 Group According to Blood Grouping

**Figure 4:**
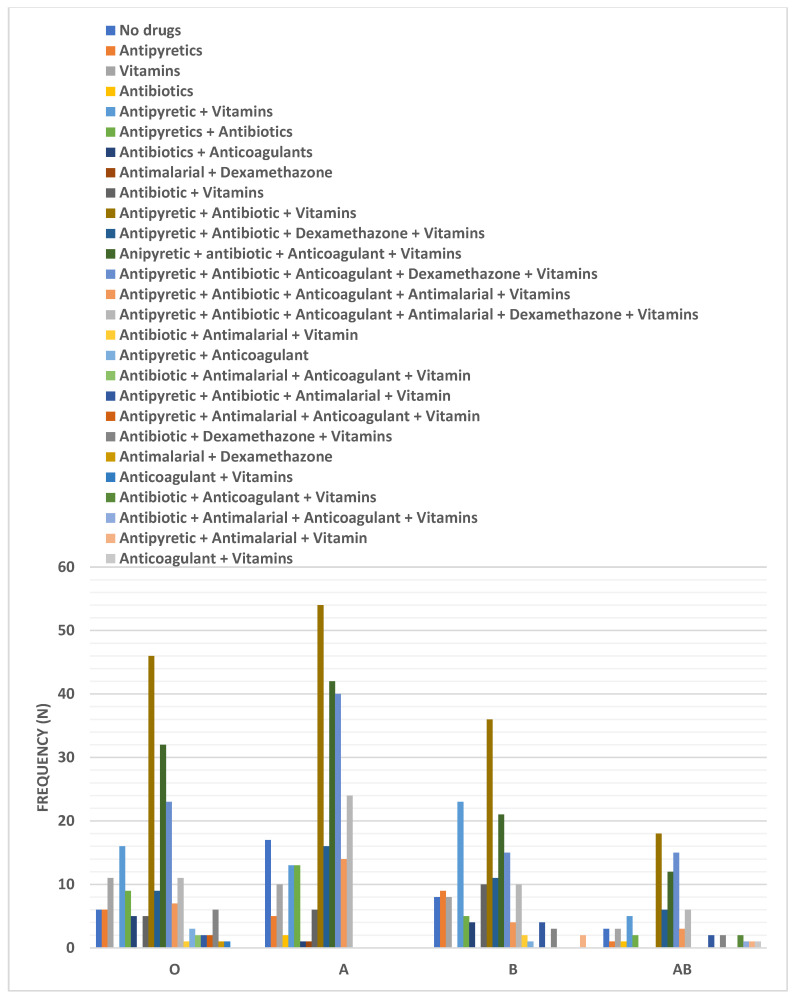
Distribution of different treatment regimens among the COVID-19 group according to the blood grouping. Data were presented as frequency (number).

Figure 4 presents the different therapeutic regimens utilized for COVID-19 viral infection treatment and their distribution among patients with respect to their blood group type. Briefly, the prevailing treatment regimen in treating COVID-19 infection included an antipyretic, antibiotic, and vitamins. The percentages of patients that used the previous regimen, with blood groups O, A, B, and AB, were 22.5%, 20.9%, 20%, and 22.5%, respectively. The second treatment regimen was similar to the previously mentioned one with the addition of an anti-coagulant. The percentages of patients who used the second regimen were 15.7%, 16.3%, 11.7%, and 14.3%, having blood groups O, A, B, and AB, respectively. The third commonly used regimen contained dexamethasone in addition to the drugs used in the second regimen. The percentages of patients who used the last regimen were 11.3%, 15.5%, 8.3%, and 17.9%, with blood types O, A, B, and AB, respectively. Pearson chi-squared analysis revealed an insignificant difference among the distribution of different treatment regimens among the COVID-19 group according to the blood grouping (*p* = 0.6328).

### 3.6. Correlation of Different COVID-19 Symptoms, the Need for Hospitalization, Recovery Time, PCR Results, and ABO Blood Grouping

The results presented in Table 2 showed no correlation between ABO blood groups and the display of high body temperature, headache, shortness of breath, cough, bone ache, GI symptoms, need for hospitalization, as well as the results of the diagnostic smear analysis (PCR) in COVID-19 patients, according to Pearson chi-squared analysis. On the other hand, Pearson chi-squared test analysis showed the existence of significant differences among ABO blood groups regarding the distribution of oxygen saturation percentage, myalgia, and recovery time after COVID-19 infection (*p* < 0.01, 0.01, and 0.05, respectively).

**Figure 5:**
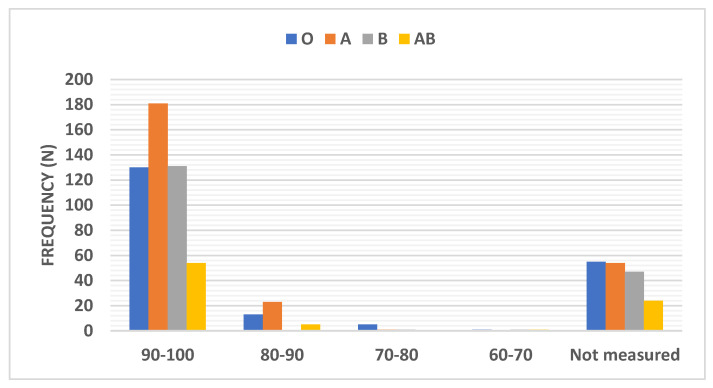
Distribution of the oxygen saturation percentage among COVID-19 patients according to the ABO blood group type. Data were presented as frequency (number).

The percentage of distribution of oxygen saturation among COVID-19 patients according to the ABO blood group type is shown in Table 2 and graphically presented in Figure 5. Among the different blood groups, blood group A showed the highest percentage of patients (70.2%) who experienced an oxygen saturation in the range of 90–100% (standardized residuals of 1.95 and Bonferroni corrected *p*-value = 0.05). The blood group B showed the lowest percentage of patients (0%) who experienced an oxygen saturation range of 80–90% (standardized residuals of 3.16 and Bonferroni corrected *p*-value = 0.0015). In addition, the blood group O showed the highest percentage of patients (1.8%) who experienced an oxygen saturation range of 70%-80% (standardized residuals of 2.24 and Bonferroni corrected *p*-value = 0.025).

**Figure 6:**
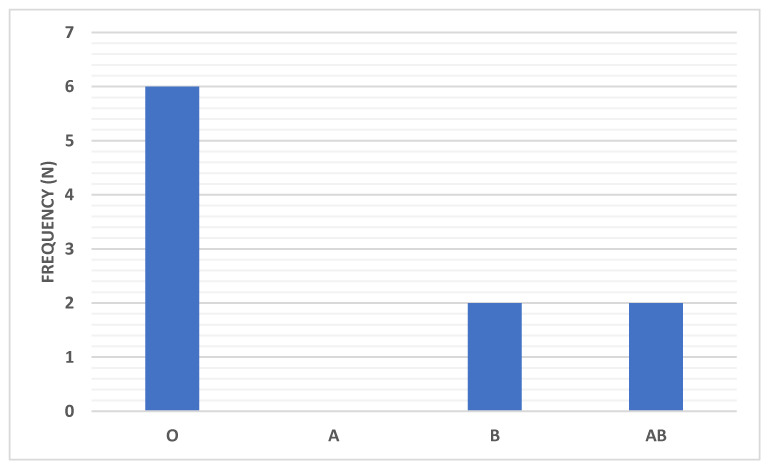
Distribution of the artificial respiration among COVID-19 patients according to the ABO blood group type. Data were presented as frequency (number).

The artificial respiration distribution among COVID-19 patients according to the ABO blood group type is shown in Table 2 and Figure 6. Among the different blood groups, the blood group O showed the highest percentage of patients (2.9%) who required artificial respiration (standardized residuals of 1.9 and Bonferroni corrected *p*-value = 0.05). On the other hand, patients with blood group A showed the lowest percentage of patients (0%) who required artificial respiration (standardized residuals of 1.89 and Bonferroni corrected *p*-value = 0.05).

**Figure 7:**
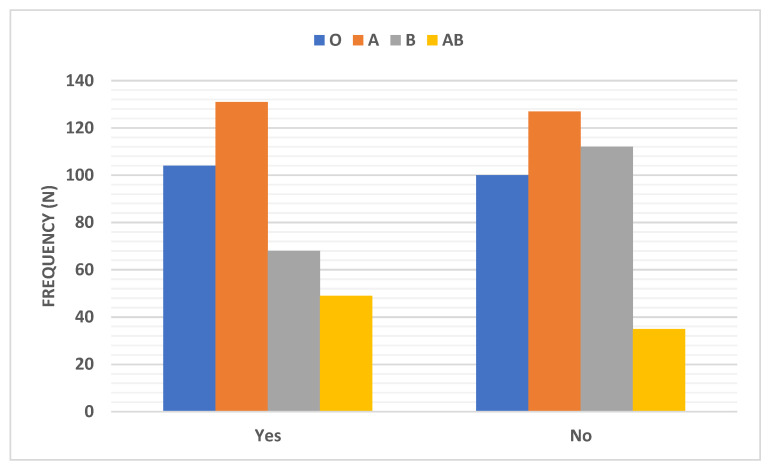
Distribution of myalgia among COVID-19 patients according to the ABO blood group type. Data were presented as frequency (number).

Myalgia distribution among COVID-19 patients according to the ABO blood group type was shown in Table 2 and Figure 7. With the different blood groups, blood group B showed the lowest percentage of patients (37.8%) who experienced myalgia (standardized residuals of 2.06 and Bonferroni corrected *p*-value = 0.039).

**Figure 8:**
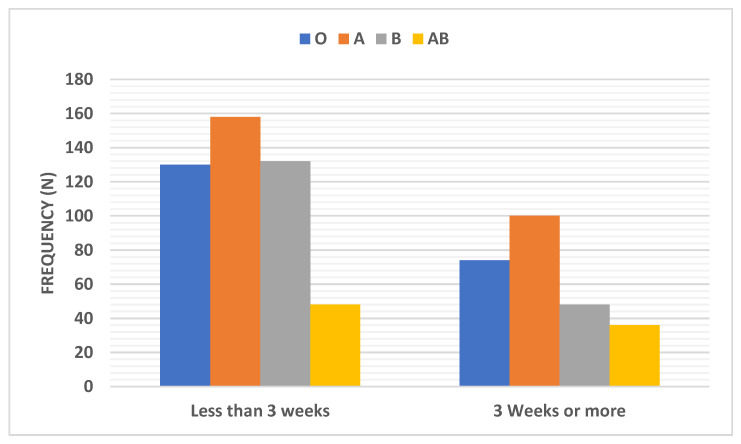
Distribution of recovery time among COVID-19 patients according to the ABO blood group type. Data were presented as frequency (number).

Recovery time distribution among COVID-19 patients according to the ABO blood group type was shown in Table 2 and Figure 8. Patients with blood group B exhibited the lowest percentage (26.7%) of patients who needed 3 weeks or more to recover compared to other blood types (standardized residuals of 2.0 and Bonferroni corrected *p*-value = 0.045).

### 3.7. Outcomes of Some Research Studies Which Document the Effect of ABO Blood Grouping on the Risk of COVID-19 Infection and Severity in Comparison with the Present Study Outcomes

Table 3 presents the findings of researchers from Spain, France, Turkey, China, and Sudan, who report that having type A blood is a high risk factor for COVID-19 infection. On the other hand, researchers from Spain, France, Canada, Turkey, China, Denmark, Sudan, and Iran reported that having type O blood represents a lower risk factor for COVID-19. Concomitantly with previous research, a meta-analysis study documents that blood type A is a positive risk factor, where blood type O is a negative risk factor. Studies done in Iraq and Iran show that AB blood is more susceptible to COVID-19 infection. Other studies performed in Spain (SCPT, severe COVID-19 plasma transfusion), France, and the USA show that the ABO blood groups are not associated with the risk of COVID-19 infection.

One study carried out in Italy and Spain showed that individuals with A+ blood type are at increased risk of respiratory failure while those with O type are at lower risk. Another research project performed in Spain showed that mild COVID-19 plasma donors (MCPD) with blood type A are at high mortality risk while those with blood type O are at low mortality risk. A study done in France showed that those with blood type O are at increased demand for O2 therapy and ICU care. On the other hand, other studies in France, Canada, China, and Iraq showed that type A blood causes unfavorable outcomes. Studies carried in Canada and India showed that AB and B blood are associated with a severe course of COVID-19 infection (Table 3).

Our study findings are in line with the studies carried in France (patients with blood type O required more O2 and ICU), Canada (patients with blood type O were less infected with a severe illness), Turkey (patients with blood type O were less infected and those with blood type A were less associated with mortality), Spain, China, Denmark, Sudan, and Iran (patients with blood type O were less infected) (Table 3).

## 4. Discussion

The main results of this observational study showed a significant difference among the allocation of different ABO blood groups in both the control group and the COVID-19 group (*p* < 0.01). In comparison to the control group, the percentage of the individuals with blood group O in the COVID-19 group was significantly lower (28% versus 36%) (*p* < 0.05), while the percentage of the individuals with blood group A was insignificantly higher (35% versus 29% respectively). This result showed that the subjects with blood group type O are the least likely to be infected with the COVID-19 virus, while subjects having blood group type A are not likely to be the most susceptible. Furthermore, the present study results offered that among the different blood groups, the blood group A showed the highest percentage of patients (70.2%) who experienced an oxygen saturation range of 90–100% (*p* < 0.05). The blood group B showed the lowest percentage of patients (0%) who experienced an oxygen saturation range of 80–90% (*p* < 0.01). In addition, the blood group O showed the highest percentage of patients who experienced an oxygen saturation range of 70–80% (*p* < 0.05). These results confirmed that among all the blood types, blood group O patients showed the highest percentage of patients (2.9%) who needed artificial respiration (*p* < 0.05). Nevertheless, blood group A patients did not need artificial respiration (0%), (*p* < 0.05).

The present study findings are in line with the studies carried in France (patients with blood type O required more O2 and ICU) [17,18], Canada (patients with blood type O were less infected with a severe illness) [20], Turkey (patients with blood type O were less infected and those with blood type A less associated with mortality) [22], Spain [16], China [24], Denmark [27], Sudan [30], and Iran [31] and the metanalysis carried out by [32] (patients with blood type O were less infected).

This study’s findings contradict the studies carried in Italy and Spain (patients with blood group A+ were at a high risk of respiratory failure while those with blood type O were protected) [15], Spain (group A patients had a high mortality risk, group O patients a low mortality) [16], France (blood group A patients had a high risk of both infection and severe course) [19], Canada (patients with blood groups A and AB showed more severe illness) [21], Turkey (blood group A patients had higher risk, group O patients had lower risk, and ABO does not affect the severity) [23], China (blood group A patients are had high risk of severity and blood group O patients had low) [24], China (blood group A patients had a high risk of infection) [25], USA, and Denmark (no association between ABO, infection, and severity) [26,27], Iraq (blood group A patients are at increased risk of both infection and severity) [28], and India (blood group O patients have decreased mortality; blood group B patients increased mortality) [29].

Our results are in accordance with previous data related to blood group O, although there is controversy with the data for blood group A. According to Wu et al. [14], type A blood imposed a higher risk of severe COVID-19, while type O blood offered protection against infection. Similarly, researchers from both China and USA reported that having type A blood is a high risk factor for COVID-19 infection, whereas having type O represents lower risk [13]. Zietz and Tatonetti [33] also reported negative harmonious relation between the O blood group and exposure to COVID-19. They did not recognize any significant association between blood group and either the need for intubation or COVID-19 related mortality, possibly due to their low size sample. In another study, Zeng et al. [34] identified higher odds of COVID-19 exposure in subjects belonging to the A blood group. In their study, the authors reported that individuals bearing blood group O exhibited reduced susceptibility to severe COVID-19, however, they could not find out if having O blood group offered protection against the virus. In a large multi-institutional retrospective review, the authors concluded that there is no relation between blood type and risk of severity progression, need for intubation, or mortality [35]. These data contradicted the results of Zhao et al. [13] who evaluated the association between blood type and death incidence in the Wuhan experience. The possible interpretation for the divergence of the observations is the racial, regional, and possible genetic variations among individuals of the same blood type. Some unrecognized blood proteins might play an essential role for COVID-19 severity of infection besides ABO blood antigens.

Subjects with certain ABO blood group types are more prone to various infection types [36]. Groot et al. [11] observed that people with blood type O are more prone to hypertension, while those bearing A, B, and AB blood types have higher susceptibility to thrombosis and myocardial infarction. In addition, Stowell and Stowell [12] observed that individuals possessing the A antigen are also predisposed to a higher risk of thromboembolism and metabolic disorders. Blood group antigens have been shown to be effective receptors for several infectious microorganisms. The virus spike (S) protein binding to the specific ABO glycan antigen receptors may support virus entry during infection [36].

Guillon et al. [37] reported particular inhibition of the adsorption of SARS-CoV S protein-expressing cells to angiotensin-converting enzyme 2 (ACE2)-expressing cell lines by anti-A antibodies. Considering the analogy between the nucleic acid sequence and ACE-2 binding capability of the aforementioned virus and the novel coronavirus [38,39,40], the observed variation in susceptibility of blood groups A and O for COVID-19 could be related to the existence of natural anti-blood group antibodies in the blood, especially the anti-A antibody in case of O blood group.

A genome-wide association study reported that blood type O subjects have an increased interleukin 6 (IL-6) level than other blood types do [41]. IL-6 is a proinflammatory cytokine promoting the release of acute-phase proteins like C-reactive protein. This hypothesis may suggest the disadvantage of being a blood type O carrier and may explain the bad prognosis of type O blood group patients that really needed artificial respiration. Higher serum interleukin-6 concentrations in COVID-19 infected subjects are suggested as a predisposing factor in almost all severe cases of the disease and the necessity for intensive care [42].

The spike COVID-19 glycoproteins express A and/or B glycan antigens, reflecting the ABO phenotype of the cells where viruses are produced. The COVID-19 viruses produced in individuals of groups A, B, AB, and O express A, B, A and B antigens, and none, respectively. People in groups A, B, AB, and O have anti-B, anti-A, none, and anti-A/anti-B/anti-A,B antibodies, respectively [43]. Anti-A, Anti-B, and Anti-A,B antibodies may decrease an individual’s chance of infection from the SARS-CoV-2 virus, resulting in a lower susceptibility of type O individuals. Blockage may be complete or not. However, once the infection is established, individuals produce viruses of their own ABO types, and the Anti-A, Anti-B, and/or Anti-A,B antibodies they possess may no longer neutralize newly produced viruses. Blood type O individuals may have a lower risk of viral infection, but any type O SARS-CoV-2 viruses they produce may infect their own cells as well as individuals with any ABO phenotypes [44]. Anti-A, Anti-B, and/or Anti-A,B antibodies may inhibit the interaction between the viral spike glycoproteins and cellular ACE2 receptors. This may prevent the entry of the SARS-CoV-2 virus into cells and neutralize the virus in a complement-dependant manner. It may also promote cytotoxic T cells. Acquisition of immunity against other viral antigens may follow [44]. In this study, the most common symptoms among COVID-19 patients were fever (63.2%), generalized weakness (51.2%), cough (51.2%), bone ache (48.3%), and myalgia (44.6%). These symptoms were in accordance with those reported in previous studies [1,45,46,47,48]. Respiratory symptoms have been demonstrated by most COVID-19 patients in previous studies [1,46,49]. In the present study, 41.6% of the patients suffered from shortness of breath, while runny nose and sneezing were demonstrated in 29.6% and 25.6% of patients, respectively. In other studies, digestive symptoms were not common [1,46], while in the present study gastrointestinal disorders were experienced by 47.7% of the patients, revealing increased likelihood of the occurrence of gastrointestinal symptoms. This result is consistent with what has been reported by Zhan et al. [50], who found that 61.2% of patients had digestive tract-associated symptoms. Evidence supporting the fecal transmission of SARS-CoV-2 and its binding capacity to ACE2 of the GI tract has been previously reported [51,52]. The prevalence of GIT symptoms over respiratory ones might be ascribed to reduced virulence with raised infectivity and changed organ susceptibility owing to virus mutations [53]. Less common symptoms were sweating (39.4%) and loss of smell and taste (32%). There were (20.8%) subjects who reported other symptoms (not mentioned) and 18 (2.5%) subjects stated that they were asymptomatic. Accordingly, cautionary measures should be applied for COVID-19 suspected subjects who had regular body temperatures and went to different outpatient clinics because of non-respiratory manifestations [53,54].

In the present study, there is an insignificant difference between the distribution of Rh type in both the control group and the COVID-19 group (*p* = 0.1913). Conversely, Zietz and Tatonetti [33] suggested a significant association between COVID-19 exposure and A and O blood groups with positive Rhesus factor (A+ and O+) solely.

The results of our study showed that most COVID-19 patients needed one to two weeks to recover from their symptoms (24.2%, and 31.1%, respectively). Furthermore, a high percentage of patients may require more than a month to recover from symptoms (16%). Some patients needed up to 3 weeks or a month to recover (11.7%, and 7.5%, respectively). Small percentages of patients required a day (1%), two (1.6%), or three days (6.8%) to recover.

The results of this work showed no correlation between ABO blood groups and the distribution of the body temperature, headache, shortness of breath, cough, bone ache, GI symptoms, and need for hospitalization. On the other hand, there were significant differences in the distribution of oxygen saturation percentage, myalgia, and recovery time after COVID-19 infection, and ABO blood groups in COVID-19 patients (*p* < 0.01, 0.01, and 0.05, respectively). Regarding myalgia, the blood group AB patients showed the lowest percentage of cases (37.8%) who experienced it (*p* < 0.05) compared to patients with other blood types. Blood group B patients also showed the lowest percentage of individuals (26.7%) who needed a long time to recover, 3 weeks or more (*p* < 0.05)

## 5. Conclusions

The results of this research showed that there is an association between ABO blood grouping and infection with the COVID-19 virus. The blood group O may be the least likely to be infected with the COVID-19 virus, whereas blood group A is not the highest. There was no association between the Rh type and the risk of COVID-19 infection. There was a positive association between the ABO blood groups and O2 saturation ratio, need for artificial respiration, myalgia, and recovery period. Patients with blood group O showed the lowest oxygen saturation ratio and the highest need for artificial respiration. Patients with blood group B were the lowest to suffer myalgia and the fastest to recover.

## Figures and Tables

**Table 1:** Demographic characteristics and blood grouping of the study population.

	Frequency, *n* (%)		
	Control (*n* = 707)	COVID-19 (*n* = 726)	Chi-Square *p* Value
Nationality			
Egyptians	699 (98.9%)	700 (96.4%)	0.0007 ***
Saudi	2 (0.3%)	20 (2.8%)	
Other	6 (0.8%)	6 (0.8%)	
Gender			
Male	117 (16.5%)	110 (15.2%)	0.4689
Female	590 (83.5%)	616 (84.8%)	
Age (year)			
<20	38 (5.4%)	9 (1.3%)	0.0002 ***
20–40	565 (79.9%)	596 (82.1%)	
40–60	90 (12.7%)	104 (14.3%)	
>60	14 (2%)	17 (2.3%)	
PCR Result			
Positive		278 (38.3%)	
Negative		157 (21.6%)	
Not done		291 (40.1)	
Type of Blood Group			
O	253 (36%)	204 (28%)	0.0096 **
A	207 (29%)	258 (35%)	
B	163 (23%)	180 (25%)	
AB	84 (12%)	84 (12%)	
Type of Rh			
Negative	113 (16%)	135 (18.6%)	0.1913
Positive	594 (84%)	591 (81.4%)	

Data were presented as frequency (number) and percentage (%). The significance between groups was determined using the chi-square test, and a *p*-value of less than 0.05 was considered significant. *** Significance at *p* ≤ 0.001; ** Significance at *p* ≤ 0.01.

**Table 2:** Correlation of different COVID-19 symptoms: need for hospitalization, recovery time, diagnostic smear (PCR) results, and ABO blood grouping system.

	Frequency, *n* (%)				
	O (*n* = 204)	A (*n* = 258)	B (*n* = 180)	AB (*n* = 84)	Chi-Square *p* Value
Body Temperature (°C)					
37–38	86 (42.2%)	110 (42.6%)	84 (46.7%)	32 (38.1%)	0.3765
38–39	90 (44.1%)	96 (37.2%)	65 (36.1%)	33 (39.3%)	
39–40	21 (10.3%)	45 (17.5%)	24 (13.3%)	17 (20.2%)	
40–41	7 (3.4%)	7 (2.7%)	7 (3.9%)	2 (2.4%)	
Headache					
Yes	128 (62.7%)	150 (58.1%)	101 (56.1%)	57 (67.9%)	0.2300
No	76 (37.3%)	108 (41.9%)	79 (43.9%)	27 (32.1%)	
Oxygen Saturation (%)					
90–100	130 (63.7%)	181 (69.9%)	131 (72.8%)	54 (64.3%)	
80–90	13 (6.4%)	23 (8.9%)	0.0 (0.0%)	5 (5.9%)	0.0029 **
70–80	5 (2.5%)	1 (0.4%)	1 (0.6%)	0.0 (0.0%)	
60–70	1 (0.5%)	0.0 (0.0%)	1 (0.6%)	1 (1.2%)	
Not measured	55 (26.9%)	54 (20.5%)	47 (26.1%)	24 (28.6)	
Artificial respiration					
Yes	6 (2.9%)	0 (0%)	2 (1.1%)	2 (2.4%)	0.0462
No	198 (97.1%)	258 (100%)	178 (98.9%)	82 (97.6%)	
Shortness of Breath					
Yes	85 (41.7%)	117 (45.3%)	63 (35%)	35 (41.7%)	0.1946
No	119 (58.3%)	141 (54.7%)	117 (65.5%)	49 (58.3%)	
Cough					
Yes	103 (50.5%)	136 (52.7%)	88 (48.9%)	38 (45.2%)	0.6576
No	101 (49.5%)	122 (47.3%)	92 (51.1%)	46 (54.8%)	
Myalgia					
Yes	104 (51%)	131 (51%)	68 (37.8%)	49 (58.3%)	0.0057 **
No	100 (49%)	127 (49%)	112 (62.2%)	35 (41.7%)	
Bone ache					
Yes	96 (47.1%)	120 (46.5%)	66 (36.7%)	43 (51.2%)	0.0742
No	108 (52.9%)	138 (53.5%)	114 (63.3%)	41 (48.8%)	
GIT Symptoms					
Yes	104 (51%)	124 (48%)	83 (46%)	41 (49%)	0.8166
No	100 (49%)	134 (52%)	97 (54%)	43 (51%)	
Hospitalization					
Yes	16 (7.8%)	18 (7.0%)	15 (8.3%)	6 (7.1%)	0.9556
No	188 (92.2%)	240 (93.0)	165 (91.7%)	78 (92.9%)	
Recovery Time					
Less than 3 weeks	130 (63.7%)	158 (61.2%)	132 (73.3%)	48 (57.1%)	0.0248 *
3 Weeks or more	74 (36.3%)	100 (38.8%)	48 (26.7%)	36 (42.9%)	
PCR Result					
Positive	70 (34.3%)	110 (42.6%)	69 (38.3%)	30 (35.7%)	0.1571
Negative	43 (21.1%)	42 (16.3%)	43 (23.8)	20 (23.8%)	
Not done	91 (44.6%)	106 (41.1)	68 (37.8)	34 (40.5%)	

Data were presented as frequency (number) and percentage (%). The significance between groups was determined using the chi-square test, and a *p*-value of less than 0.05 was considered significant. ** Significance at *p* ≤ 0.01; * Significance at *p* ≤ 0.05.

**Table 3:** Outcomes of some research studies which document the effect of ABO blood grouping on the risk of COVID-19 infection and severity and its comparison with the present study outcomes.

Study	Location	Number of COVID-19 Patients	ABO and Risk of COVID-19 Infection	ABO and COVID-19 Severity	Drawbacks	Agreement with the Present Study Results	
						Infection	Severity
Ellinghaus et al. [15]	Italy Spain	1980 ICU		GroupA+ at higher risk of respiratory failure, group O at lower risk			No
Muñiz-Diaz et al. [16]	Spain	854 MCPD 956 SCPT	MCPD Group A at higher risk Group O at lower risk SCPT No association	MCPD Group A high mortality risk, group O low mortality SCPT No association		Yes O less	No
Boudin et al. and Flegel, [17,18]	France	1279	No association between ABO and Rh blood groups and risk of COVID-19 infection	Concerning O2 therapy requirement, 5 patients were group A; 5 group B, and 10 group O The patients admitted to the ICU were 1 group B and 2 group O	Missed blood groups of 16 patient	No	Yes More group O patients required O2 and ICU
Kibler et al. [19]	France	19 TAVR	Group A at higher risk	Group A showed unfavorable outcomes		No	No
Ray et al. [20]	Canada	225 556	Group O− at lower risk	Group O− may be associated with severe illness		Yes O less	Yes O more
Hoiland et al. [21]	Canada	22 ICU		Groups A or AB showed more required mechanical ventilation, CRRT, and prolonged ICU admission than groups O or B			No
Solmaz and Araç, [22]	Turkey	1667	Group A at higher risk Group O at lower risk	Group A does not affect the course of the disease and is not associated with mortality		Yes Group O less	Yes Group A did not have increased mortality
Göker et al. [23]	Turkey	207	Group A at higher risk Group O at lower risk	ABO groups do not influence clinical outcome		No	No
Li et al. [24]	China	3959	Group A at higher risk Group O at lower risk	Group A was at higher risk of hospitalization, and group O had a lower risk		Yes Group O less	No
Fan et al. [25]	China	105	Group A (females) at higher risk		Small sample size	No	
May et al. [26]	USA	165	No association between ABO and Rh blood groups and risk of COVID-19 infection	No association between blood group and severity	Small sample size	No	No
Barnkob et al. [27]	Denmark	7422	Group O at low risk	No association between ABO blood group and the risk for hospitalization or death		Yes Group O less	No
Ad’hiah et al. [28]	Iraq	300	Group AB at higher risk	Group A may be associated with an increased risk of death		No	No
Padhi et al. [29]	India	8452		Group O associated with a decreased mortality rate, group B associated with an increased mortality rate			No
Taha et al. [30]	Sudan	557	Group A+ at higher risk Group O+ at lower risk			Yes Group O less	
Abdollahi et al. [31]	Iran	397	Group AB at higher risk Group O at lower risk			Yes Group O less	
Golinelli et al. [32]	Meta-analysis heterogenous population	7503	Group A at higher risk Group O at lower risk		Considerable heterogeneity found in the study population and the online date, which are still preliminary	Yes O less	

ICU: Intensive care units; TAVR: Transcatheter aortic valve replacement; MCPD: Mild COVID-19 plasma donor; SCPT: Severe COVID-19 plasma transfusion.

## Data Availability

All relevant data is available in the present manuscript. The data presented in this study are available on request from the corresponding author.
